# Metabolic Syndrome and Psoriasis: Pivotal Roles of Chronic Inflammation and Gut Microbiota

**DOI:** 10.3390/ijms25158098

**Published:** 2024-07-25

**Authors:** Paola Secchiero, Erika Rimondi, Annalisa Marcuzzi, Giovanna Longo, Chiara Papi, Marta Manfredini, Matteo Fields, Lorenzo Caruso, Roberta Di Caprio, Anna Balato

**Affiliations:** 1 Department of Translational Medicine and LTTA Centre, University of Ferrara, 44121 Ferrara, Italy; paola.secchiero@unife.it (P.S.); erika.rimondi@unife.it (E.R.); 2 Department of Translational Medicine, University of Ferrara, 44121 Ferrara, Italy; giovanna.longo@unife.it (G.L.); chiara.papi@unife.it (C.P.); marta.manfredini@edu.unife.it (M.M.); matteo.fields@unife.it (M.F.); 3 Department of Environmental and Prevention Sciences, University of Ferrara, 44121 Ferrara, Italy; lorenzo.caruso@unife.it; 4 Dermatology Unit, Department of Mental and Physical Health and Preventive Medicine, University of Campania Luigi Vanvitelli, 80131 Naples, Italy; rob.dicaprio@gmail.com (R.D.C.); anna.balato@unicampania.it (A.B.)

**Keywords:** metabolic syndrome, inflammation, insulin resistance, adipose tissue, psoriasis

## Abstract

In recent years, the incidence of metabolic syndrome (MS) has increased due to lifestyle-related factors in developed countries. MS represents a group of conditions that increase the risk of diabetes, cardiovascular diseases, and other severe health problems. Low-grade chronic inflammation is now considered one of the key aspects of MS and could be defined as a new cardiovascular risk factor. Indeed, an increase in visceral adipose tissue, typical of obesity, contributes to the development of an inflammatory state, which, in turn, induces the production of several proinflammatory cytokines responsible for insulin resistance. Psoriasis is a chronic relapsing inflammatory skin disease and is characterized by the increased release of pro-inflammatory cytokines, which can contribute to different pathological conditions within the spectrum of MS. A link between metabolic disorders and Psoriasis has emerged from evidence indicating that weight loss obtained through healthy diets and exercise was able to improve the clinical course and therapeutic response of Psoriasis in patients with obesity or overweight patients and even prevent its occurrence. A key factor in this balance is the gut microbiota; it is an extremely dynamic system, and this makes its manipulation through diet possible via probiotic, prebiotic, and symbiotic compounds. Given this, the gut microbiota represents an additional therapeutic target that can improve metabolism in different clinical conditions.

## 1. Metabolic Syndrome as a Multifactor Condition: Incidence and Diagnostic Criteria

Metabolic syndrome (MS) is the most common non-communicable disease and affects almost 30% of the population worldwide, significantly impacting public health costs [1,2]. The increased incidence of this pathology has been linked to different factors, such as a sedentary lifestyle, consumption of a diet that is rich in fat and red meat and poor in fiber, and circadian dysrhythmia [2,3].

The pathophysiology of MS has been hypothesized to involve different mechanisms that have not been fully clarified yet, and over the years, the classification of MS has evolved with regard to its diagnostic definitions [4]. In 2009, it was established that the presence of at least three out of five risk factors is necessary for a diagnosis of MS [5,6]. These risk factors have specific cut-off values: waist circumference (threshold are different for population, country and gender); triglycerides ≥150 mg/dL; HDL-C < 40 mg/dL in males and <50 mg/dL in females; systolic blood pressure ≥ 130 mm Hg and/or diastolic blood pressure ≥85 mm Hg; fasting glucose ≥100 mg/dL.

MS does not manifest suddenly; instead, it results as a consequence of a progressive process that, starting from the alteration of a single metabolic parameter, eventually results in the simultaneous alteration of multiple factors [7]. Physiopathological changes in MS are correlated with hormonal changes, aging [1], prothrombotic and proinflammatory states, and liver diseases, such as non-alcoholic fatty liver disease (NAFLD) [8].

MS is characterized by a set of abnormal physiological parameters that are considered to be cardiometabolic risk factors, including abdominal obesity, insulin resistance, impaired glucose metabolism, diabetes, arterial hypertension, and atherogenic dyslipidemia [1].

The presence of one or more of these abnormal parameters is correlated with a significant increase in the risk of ischemic heart disease and stroke [9].

These components are part of a larger cluster of factors that indicate a risk for pathologies of the cardiovascular system, and these are categorized into predisposing, major, and emerging factors.

The predisposing risk factors for cardiovascular diseases are obesity, especially an increase in visceral fat, low levels of physical activity, and an atherogenic diet [10,11,12]. The major risk factors for cardiovascular diseases are cigarette smoking, high blood pressure, increased concentrations of low-density lipoprotein (LDL) cholesterol, reduced levels of high-density lipoprotein (HDL) cholesterol, a family history of coronary heart diseases, and age [13,14]. Emerging risk factors include an increased level of triglycerides, the presence of small dense LDL, insulin resistance, glycemic intolerance, and pro-inflammatory and prothrombotic states [14,15,16,17].

## 2. Metabolic Syndrome Is Related to Adipose Tissue, Insulin, and Immunity

Numerous studies have shown that inflammation alters insulin secretion through different aspects related to energy metabolism. The inflammation state involved in these processes differs from other purely inflammatory diseases, as it involves immune activation that is persistent over the years but occurs in a subacute and silent form. Inflammation in the adipose tissue begins when adipocytes are stressed due to increased fatty acid intake. When their cytoplasm is engulfed with lipid droplets, they begin losing their physiological functions, secreting pro-inflammatory cytokines and transforming into senescent cells. The immune system is activated by this tissue inflammation and attempts to promote tissue repair by eliminating senescent adipocytes and disposing of excessive triglycerides; macrophages accumulate lipid droplets, but this renders them dysfunctional, as they lose their physiological role. Senescent adipocytes also release factors that slowly turn immune cells into senescent cells, an event called immunosenescence. Senescent immune cells and lipid-engulfed macrophages continue to produce proinflammatory cytokines, impairing the production of pro-resolving factors, leading to chronic, low-grade tissue inflammation [18]. In addition, with time, the continuous production of numerous proinflammatory factors also causes systemic inflammation by affecting various organs such as fatty tissue, the pancreas, the liver, muscles, the brain, and the heart.

Cytokines and acute-phase proteins mark the immunoinflammatory response that accompanies MS; C-reactive protein is one of the molecular markers whose levels are already higher in pre-diabetic patients [19,20]. The levels of specific cytokines increase progressively with the worsening of glycemic intolerance, and this correlation indicates the onset of a dangerous feedback loop between the inflammatory response and the metabolic alterations that support insulin resistance [21,22].

### 2.1. Chronic Inflammation Is a Major Trigger for Metabolic Diseases

**Figure 1:**
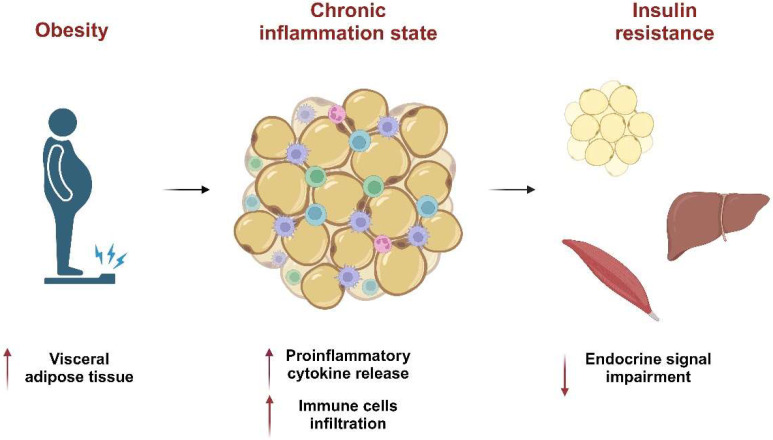
Obesity is characterized by a chronic inflammation state that induces insulin resistance. The arrows pointing upwards indicate an increase, while the arrows pointing downwards indicate a decrease. Image created with BioRender.com.

Chronic inflammation is a key aspect of obesity and diabetes, diseases in which insulin, a pancreatic hormone, can no longer effectively perform its functions in adipose tissue, muscles, and the liver. Insulin resistance is caused by the enlargement of adipose tissue cells and the increased release of pro-inflammatory substances such as tumor necrosis factor (TNF)-α and interleukin (IL)-6, together with progressive adipose tissue infiltration by immune cells [23,24] (Figure 1).

TNF-α, which is overexpressed by adipocytes under conditions of insulin resistance and the hypertrophy of adipose cells, causes insulin resistance by interfering with the transmission of the insulin signal and activating inflammation processes. Studies performed on a knockout (KO) TNF mouse model have shown that the removal of this factor improved glucose tolerance and increased insulin sensitivity. Moreover, some polymorphisms of the TNF gene in humans have been associated with the development of obesity, type 2 diabetes mellitus (T2DM), and metabolic syndrome [25,26].

Another important immunomodulatory agent is IL-6, which plays an active role in the regulation of acute-phase reactions, the activation of T helper cells, the inhibition of regulatory T cells (Tregs), and the differentiation of B lymphocytes. The main cellular sources of IL-6 are monocytes and T cells, but it can also be produced by other cells infiltrating adipose tissue. IL-6 plays a role in the recruitment of neutrophils and macrophages and is also associated with the pathogenesis of chronic inflammatory disease [27,28,29].

Increased levels of TNF-α and IL-6 cause the activation of inflammatory processes; additionally, both cytokines are insulin-resistance inducers at the peripheral level and hepatic level, respectively.

It is generally recognized that changes in nutrient availability, such as lipids, glucose, or amino acids, also affect cellular processes and lead to immune cell activation. This mechanism, called immunometabolism, identifies the balance between the immune system’s efficiency and its energy needs [30,31,32]. In the presence of an energy shortage, the immune response can be ineffective, unable to neutralize antigens, or even harmful, with an exaggerated inflammatory reaction that activates oxidative processes that damage cellular structure and functions. An example of this mechanism is how free fatty acids act as ligands for toll-like receptor 4 (TLR4), with TLR4-free mice in one study being protected from obesity induced by a diet rich in saturated fatty acids [33]. Similarly, high levels of circulating saturated fatty acid palmitate, which were observed in mice fed a high-fat diet, directly promoted the activation of the immune response by activating inflammasomes, resulting in the release of IL-1β and IL-18 by macrophages and promoting the polarization of CD4+ lymphocytes toward an effector phenotype [34,35]. In particular, IL-1 and IL-18 are cytokines that stimulate the production of IFN-γ in T cells. IL-18 has already been identified by numerous clinical and experimental studies as one of the main factors involved in the progression and destabilization of atherosclerotic plaques; an important role is attributed to this cytokine in the process that leads to the rupture of these plaques, in the formation of thrombi, and, finally, in acute coronary syndrome [36,37,38].

### 2.2. Adipose Tissue and Insulin Play a Balancing Role in Hormonal Homeostasis and the Immune System

A network is created to regulate inflammation, insulin activity, and glucose metabolism at the local and systemic levels. Obesity induces an increase in visceral adipose tissue and contributes to the development of an inflammatory state and impaired adipocyte metabolism, which in turn produces an increase in numerous proinflammatory cytokines responsible for insulin resistance. Numerous studies have shown that an excess of adipose tissue has a pro-inflammatory effect and that low-grade chronic systemic inflammation could be considered to be a new cardiovascular risk factor [39].

In obese patients, there is an inability to biosynthesize new adipocytes capable of storing fat, which accumulates in adipocytes and other cells already present in adipose tissue, resulting in cellular dysfunction. Macrophages and lymphocytes infiltrate the hypertrophic adipose tissue, resulting in the formation of an inflammatory outbreak that interferes with the maturation of new fat cells, causing the adipose tissue to be unaffected by insulin action [40,41]. The establishment of this vicious cycle, with the involvement of inflammation, is associated with the onset of insulin resistance, which is one of the mechanisms behind the onset of T2DM.

Among the biologically active molecules involved in maintaining the balance of these physiological processes, interest has risen concerning adiponectin regulation [42]. This hormone has different active forms that perform diverse functions; single-chain adiponectin stimulates the use of fatty acids in muscle tissue, whereas forms of adiponectin with a high molecular weight are responsible for increasing insulin sensitivity. Adiponectin exerts its action by binding to two receptors, one of which (AdipoR1) is present in all cells but is particularly abundant in the muscles and liver, and AdipoR2, which is especially present in the liver. A third receptor is T-cadherin, which mediates the action of the hormone in the walls of blood vessels, smooth muscles, and the heart [43]. Adiponectin is a key hormone in processes that regulate the use of nutrients, especially glucose and triglycerides, by the entire body, and it plays a central role in regulating the insulin sensitivity of muscle tissue, also stimulating insulin production by the pancreas [44]. In the liver, this hormone reduces gluconeogenesis, increases insulin sensitivity, promotes the transport of glucose within liver cells, and, finally, stimulates fatty acid oxidation. Adiponectin also stimulates the activity of ceramidases—enzymes that convert ceramides into sphingosine. An excess of ceramides is associated with insulin resistance, cell death, inflammation, and atherosclerosis; therefore, adiponectin action is important in the regulation of ceramide content at the membrane level [45,46].

Most adiponectin action is mediated by AMPK and PPAR-α, a nuclear enzyme and a receptor, respectively, that are involved in the use of glucose and fatty acids for energy production in situations when the availability of substrates is reduced, during fasting, or when energy requirements are high.

In addition, a different isoform of the same receptor (PPAR-γ) plays a crucial role in the proper functioning of the adipocyte metabolism. It has been observed that an increased presence of inflammatory molecules in adipose tissue alters the levels of different forms of the protein PPAR-γ, reducing their functional activity and replacing them with a defective protein, PPARγΔ5. In particular, a study revealed that an increase in TNFα, secreted by hypertrophic adipocytes and macrophages recruited from the inflamed adipose tissue of patients with hypertrophic obesity, was able to disrupt the balance between PPARγΔ5 and PPARγ in favor of the defective protein. The relative increase in this abnormal protein could explain, at least in part, the defect in the formation of new adipocytes observed in patients with obesity. The PPARγΔ5 truncated form, hindering the activity of the complete protein PPARγ, interferes with the normal formation of new adipocytes and is associated with insulin resistance and diabetes in individuals with obesity [47,48].

## 3. Psoriasis and Metabolic Syndrome, a Convergent Interaction between Immunological and Metabolic Factors

Psoriasis is a chronic relapsing inflammatory skin disease affecting 2–3% of the general population worldwide [49]. There are several clinical cutaneous manifestations of Psoriasis; most commonly, it presents as chronic, symmetrical, erythematous, scaling plaques [50]. It is characterized by an increased release of pro-inflammatory cytokines and the chronic activation of innate and adaptive immunity, which results in altered keratinocyte proliferation and differentiation, as well as long-term damage to multiple tissues and organs [51]. For this reason, although it is preferentially classified as a skin disease, it is well established that Pso is associated with systemic inflammation and is associated with multiple comorbidities, such as psoriatic arthritis [52], Crohn’s disease [53], cardiovascular disease [54], and metabolic syndrome [55]. In particular, a growing number of studies and recent meta-analyses have identified a close relationship between Psoriasis and metabolic disorders, including obesity, hypertension, diabetes mellitus, hyperlipidemia, and obesity-associated NAFLD [56,57]. To date, the underlying pathways that link Psoriasis to MS are complex and not fully understood. The association is multifactorial, involving both genetic and environmental factors and often overlapping with metabolic abnormalities that frequently coexist in psoriatic patients [58]. Psoriasis is a T cell-mediated inflammatory disease characterized by the expansion and activation of Th-1, Th-17, and Th-22 cells. Once activated, these cells promote the production of a barrage of proinflammatory mediators, including but not limited to TNF-α, IL-6, IL-1, IL-17, IL-22, IL-23, vascular endothelial growth factor (VEGF), and interferon (IFN)-γ from keratinocytes, lymphocytes, and other immune cells; additionally, these cells contribute to the immunopathogenesis of skin lesions and drive systemic involvement during the course of Psoriasis [59]. Furthermore, emerging evidence suggests that the same pro-inflammatory cytokines contribute to numerous disorders within the spectrum of MS, including obesity, diabetes mellitus, hypertension, NAFLD, and hyperlipidemia [60]. Moreover, the role of tissue inflammation in fostering insulin resistance (IR) has been widely acknowledged. IR serves as the primary defect driving the onset of T2DM, and it is also a central component of MS [61]. In addition, abdominal adipose tissue accumulation, which is a key pathogenic factor of MS, is another major source of several proinflammatory cytokines in addition to adipokines [62]. Once considered an inert location of energy storage, adipose tissue has been identified by several studies over the last decade as a major secretory organ that plays a central role in a complex network of endocrine, paracrine, and autocrine crosstalk between organs and tissues such as the heart, the vasculature, the liver, muscles, the pancreas, and skin [62]. Uncontrolled adipocyte hypertrophy leads to abnormal adipokine release, with an increase in proinflammatory adipokines (leptin, visfatin) and a reduction in anti-inflammatory adipokines (adiponectin), leading to local inflammation and immune cell recruitment [63]. Higher levels of inflammatory cytokines/chemokines, including IL-6, TNF-α, IL-1β, and CCL2, are also secreted from the adipose tissue of individuals who are obese, contributing to the development of systemic inflammation [64].

In this complex scenario, IL-17, one of the most representative cytokines of Psoriasis, has emerged as a key mediator in the intricate interplay that exists between inflammation, IR, and T2DM, hinting that it could also be a mediator in bridging MS and Psoriasis [65]. Particularly, the number of IL-17- and IFN-γ-producing T cells positively correlates with adiposity [66]. These findings are consistent with observations from studies where a significant infiltration of both CD4+ and CD8+ T cells was observed in the adipose tissue of mice with obesity with respect to lean mice, predominantly characterized by a Th2- and Treg-oriented immune response [67]. Additionally, both mice with diet-induced obesity and human subjects with obesity demonstrated heightened levels of IL-6, which are predisposing factors in Th17 lineage expansion, consequently leading to an increase in the number of CD4+ cells secreting IL-17 [67]. Similarly, elevated IL-17 serum levels have been found in individuals with MS and T1DM compared to healthy controls [68]. In line with this, psoriatic patients exhibiting a robust response to Secukinumab, an anti-IL-17A monoclonal antibody, showed consistent reductions in mean body weight, waist circumference, and BMI [69]. Moreover, the combined administration of anti-IL-17A monoclonal antibodies (Secukinumab and Ixekizumab) exhibited promising outcomes, reducing fasting glucose levels in imiquimod-treated mice and ameliorating hyperglycemia in psoriatic patients [69].

Notably, TNF-α is highly expressed in both Psoriasis and MS [70]. TNF-α stimulates adipocyte leptin synthesis, induces lipolysis, and inhibits both lipogenesis and anabolic insulin-like growth factor 1 (IGF-1) production [71]. Thus, it could have a systemic role in obesity development, with mechanisms promoting dyslipidemia and body mass increase. This insight might partially explain (i) the elevated TNF-α levels observed in obese individuals and (ii) the weight loss noted in psoriatic patients undergoing treatment with TNF-α inhibitors or other antipsoriatic agents that decrease TNF-α expression levels [72]. Indeed, it has been demonstrated that treatment with Etanercept or Adalimumab improved metabolic parameters, including blood lipid and glucose levels, as well as systolic and diastolic blood pressure [73]. Another study has further confirmed that anti-TNF therapies improve the metabolic profiles of psoriatic patients by downregulating their total cholesterol and LDL levels [74]. Moreover, TNF-α also exerts effects that counteract insulin receptor activity and inhibit the glucose transporter (GLUT)-4, thereby leading to elevated insulin levels that stimulate the CNS area controlling appetite. Despite accumulating evidence implicating TNF-α in IR, anti-TNF-α therapies have not shown consistent improvements among patients who are diabetic or have IR. Similarly, in non-diabetic psoriatic patients, anti-TNF-α therapies did not significantly alter the serum concentrations of leptin and resistin [75].

Conversely, adiponectin is an adipokine endowed with anti-inflammatory, insulin-sensitizing, and antiatherogenic properties, the secretion of which is diminished by proinflammatory cytokines. Numerous investigations have underscored reduced adiponectin levels in psoriatic patients, hinting at the potential involvement of adiponectin in protection against Psoriasis. It has been reported that adiponectin inhibits both the proliferation and differentiation of keratinocytes. It also suppresses involucrin, as well as TGFβ-2 and -3 expression, and decreases IL-6, IL-8, IL-17, IL-22, and TNF-α secretion by human keratinocytes. Intriguingly, adiponectin also appears to exert a suppressive effect on IL-17 production from T cells [76]. Recent experimental studies in animal models have even suggested the possibility of preventing and potentially reversing MS through the stabilization of adiponectin levels [77].

## 4. Gut Microbiota Plays a Pleiotropic Role in the Pathogenesis of Metabolic Syndrome

A healthy microbiota is the result of the proper absorption of vitamins, minerals, and antioxidants that are essential to maintain/obtain homeostasis in an organism.

Several studies have shown that the gut microbiota has a role in MS, with causal or consequential implications. The gut microbiome is formed by microbial genomes, which encode 150 times more genes than the human genome; indeed, the term holobiont refers to biological entities that represent the sum of different contributions—10% of human cells and 90% of microbial cells [78,79]. Intestinal microorganisms perform various actions, such as hydrolysis and the fermentation of dietary polysaccharides that the human organism would not be able to digest on its own [80,81].

The main products of intestinal microbiome metabolism are monosaccharides and fatty acids, which have physiological and immune functions in the intestine, and butyrate and propionate acetate, which can be used as substrates for lipogenesis and gluconeogenesis and are also energy metabolism regulators [82,83,84]. It is a known fact that the microbiome has an important role in the fermentation of polysaccharides introduced by the diet, resulting in the intestinal absorption of nutrients and in the modulation of signals of hunger and satiety, as well as contributing to the release of hormonal substances that can affect the dynamics of IR.

At the same time, the intestinal mucosa is the primary site of microbial interaction with the immune system; therefore, the risk of triggering local and systemic inflammatory phenomena is always present.

The gut microbiota is an extremely dynamic system, and this makes its manipulation through diet possible, via probiotic, prebiotic, and symbiotic compounds; as such, it represents an additional therapeutic target that can improve metabolism in different clinical conditions [85,86]. In particular, recent clinical studies have highlighted how the manipulation of the microbiome composition with Bifidobacterium (*Bifidobacterium longum* strain BIOCC1719) could favor a reduction in the physiological risk of MS in adults who are overweight [87,88]. Moreover, since the first study in 2006, which confirmed the close relationship between the microbiota and metabolic disorders, it was evident that the microbiota influenced weight gain and obesity. Said study demonstrated how the transplantation of the intestinal microbiota from normal mice to mice deprived of bacterial flora, called Germ-Free mice, and fed a “Western diet” (rich in red meat, fats, sugars, and refined cereals/carbohydrates) resulted in significant weight increases in the transplanted mice, also leading to obesity [89]. Following this evidence, numerous studies have been conducted to verify the role of the microbiota in MS and related pathologies; all of the results have converged in defining the pleiotropic role of this entity in the pathogenesis of MS [90,91].

Furthermore, it should also be noted that some in vivo studies have pointed out that the association between the microbiome and MS might already begin in the prenatal period. The bacteria, in fact, can cross the placenta and be transferred from the mother to the fetus, colonizing the sterile intestine at birth. At the intestinal level, such bacteria would act by suppressing the expression of both FIAF (fasting-induced adipose factor), an inhibitor of LPL, and AMPK at the hepatic and muscular levels, thus leading to weight gain as a result of high carbohydrate and fat contents in the diet [92].

To date, there are a limited number of studies examining the microbial composition of patients with Psoriasis; nevertheless, an association between alterations in gut microbiota diversity, compared to healthy subjects, and the development of skin disease has been observed [93,94]. The intestinal microbiota plays a significant role in upholding epithelial barrier integrity and orchestrating mucosal immunity against external pathogens [95]. Variations in intestinal permeability have been observed between individuals with and without T2DM. Moreover, a disruption in barrier integrity has been strictly associated with the onset of MS [96]. Enhanced intestinal barrier function and mitigated metabolic endotoxemia have been shown to induce significant weight loss and ameliorate IR in mice with diet-induced obesity. Conversely, compromised intestinal barrier function can lead to bacterial translocation, resulting in the production of endotoxins or harmful metabolites that trigger systemic inflammation and exacerbate MS [97]. Thus, a compromised mucosal barrier induced by gut microbiota dysbiosis contributes to the development of MS.

Several studies have revealed that barrier integrity impairment and bacterial translocation are also implicated in the pathogenesis of Psoriasis [98]. In patients with moderate-to-severe Psoriasis, the serum markers of intestinal barrier integrity injury increased [99]. In addition, an imbalance in the two predominant phyla of the gut microbiome, *Firmicutes* and *Bacteroidetes*, has been reported in psoriatic patients, with these phyla collectively comprising 90 percent of the microbiota [100]. In their study, Chen et al. found that the ratio of *Firmicutes* to *Bacteroidetes* was disrupted in psoriatic patients compared to controls, with a reduction in *Bacteroidetes* and an elevation in *Firmicutes* [101]. Huang et al. confirmed that *Bacteroidetes* were a key factor contributing to microbiota dysbiosis in psoriatic patients, whereas *Firmicutes* were a key factor contributing to the microbiota distribution in healthy subjects [102]. Moreover, Codoñer et al. observed a distinct gut microbiome profile in psoriatic patients characterized by an increased presence of *Faecalibacterium* and a reduction in *Bacteroides*, alongside higher levels of the genera *Akkermansia* and *Ruminococcus*. However, this study involved 300 healthy controls sourced from the “Human Microbiome Project”, posing challenges in ensuring a well-matched comparison between the healthy controls and psoriatic patients regarding other potential influencing factors such as age, gender, dietary habits, and BMI, thereby potentially impacting the study’s outcomes [93]. Furthermore, Shapiro et al. also demonstrated significant increases in the phyla *Firmicutes* and *Actinobacteria* in psoriatic patients, as well as in the abundance of species such as *Ruminoccocus gnavus, Dorea formicigenerans*, and *Collinsella aerofaciens*, whereas species such as *Prevotella copri* and *Parabacteroides distasonis* were significantly depleted [103]. Therefore, intestinal barrier disruption and bacterial translocation resulting from gut microbiota dysbiosis, as well as different microbiome profiles in psoriatic patients, could be novel factors to consider in order to better understand the development of metabolic diseases in patients with Psoriasis.

**Figure 2:**
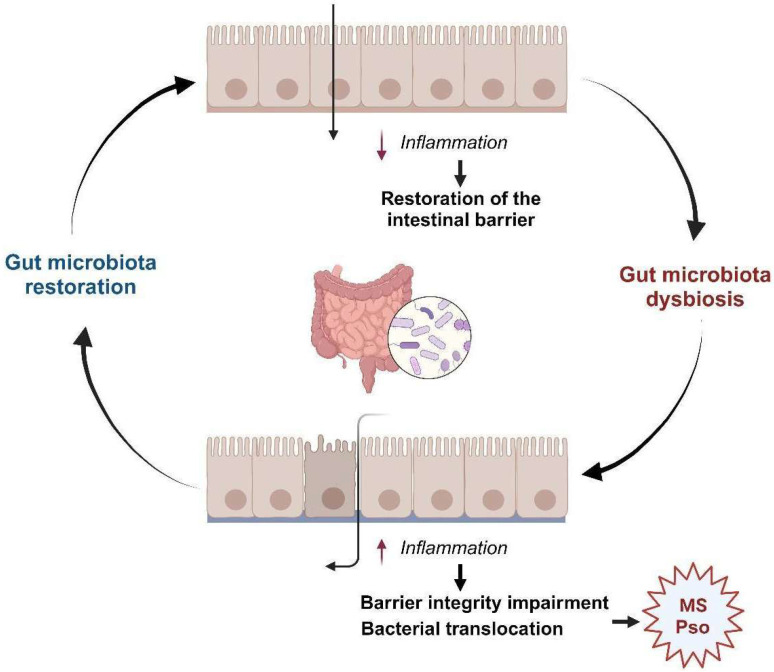
Pleiotropic role of the gut microbiome in metabolic syndrome (MS) and Psoriasis. Image created with BioRender.

Moreover, it has been demonstrated that changes in the gut microbiota can promote Th17-mediated skin inflammation. These alterations in metabolite production and anti-microbial signaling can affect immune cell activation through the IL-23/IL-17 signaling pathway, shedding light on the mechanism of increased inflammation in Psoriasis, in addition to the crucial roles of autoreactive T cells and proinflammatory cytokines, which occur in the disease. Indeed, in lesional psoriatic skin, critical cellular and molecular pathways are affected by the activation of dermal DCs that increase the proliferation of T lymphocytes, specifically T helper Th17 and Th22 in the acute phase and IFN-producing T cells in the chronic phase. T cell infiltration in active psoriatic skin creates a cytokine environment that leads to the overexpression of various inflammatory mediators and enhances local immune reactivity. For example, *Staphylococcus aureus*, belonging to the Firmicutes Phylum, produces superantigens like TSST-1 that stimulate keratinocytes and DCs to produce pro-inflammatory cytokines and IL-23, respectively. This leads to Th17 cell activation and IL-17 production, promoting psoriatic features. In the Actinobacteria phylum, *Corynebacterium striatum* directly stimulates keratinocytes and DCs to produce IL-1β, IL-6, and IL-23, enhancing Th17 cell differentiation. In the Proteobacteria phylum, *Escherichia coli* and *Neisseria mucosa* activate macrophages and DCs through LPS, triggering IL-23, IL-6, and IL-1β production, which stabilizes Th17 cells and promotes IL-17 production. Additionally, spore-forming bacteria like *Clostridia* and *Bacteroides* fragilis modulate immune responses by inducing colonic Tregs and balancing Th1/Th2/Th17 cells, while segmented filamentous bacteria (SFB) induce Th17 cell differentiation [83]. In this regard, a study by Chen et al. [92] presented compelling findings, indicating significantly greater changes in the gut microbiome of psoriatic patients treated with Secukinumab (an IL-17 inhibitor) compared to those treated with Ustekinumab (an inhibitor of IL-12 and -23). Secukinumab therapy altered the gut microbiota more significantly than Ustekinumab treatment, with these alterations including increases in the relative abundance of the phylum Proteobacteria and decreases in Bacteroidetes and Firmicutes. Following Secukinumab medication, the relative number of the families of Pseudomonadaceae, Enterobacteriaceae, and Pseudomonadales increased considerably. On the other hand, there was no significant change in gut microbiome composition after Ustekinumab treatment, and only the genus Coprococcus grew considerably after six months of Ustekinumab therapy. Furthermore, the same study found that the initial composition of the microbiome differed significantly between responders and non-responders to Secukinumab treatment [92]. Moreover, dysbiosis may increase gut permeability, leading to immune activation. Intestinal barrier function, maintained by Type 3 innate lymphoid cells and Th17 cells, is crucial for antimicrobial defense and homeostasis, influenced by microbial metabolites like Indole-3-aldehyde (IAld), which protect against *Candida albicans* colonization through IL-22 modulation (Figure 2). Thus, understanding these mechanisms offers potential targets for therapeutic interventions to modulate these pathways and alleviate Psoriasis symptoms [95]. Therefore a dysregulated gut microbiota in patients with Psoriasis may be a novel therapeutic target to alleviate MS. 

Considerable evidence has demonstrated that the microbiota plays a key role in human health and diseases. It is to be expected that these new insights will translate into diagnostic, therapeutic, and preventive measures in the context of personalized/precision medicine.

## 5. Conclusions

MS and T2DM are common conditions in old age and are related to an unhealthy lifestyle, a pro-inflammatory diet, and physical inactivity. Laboratory investigations allow for the diagnosis, evaluation, and monitoring of the extent of an inflammatory process, whether it is acute or chronic, high- or low-grade, and its evolution over time in response to therapies [104,105].

Therapeutic intervention must be combined with a targeted diet well followed by patients to allow for probiotic action at the microbiota level. A high-fiber diet could be a protective factor against obesity by influencing both intestinal fermentation and the microbiome; in fact, increased fermentation in the colon contributes to a decrease in adiposity [106,107].

Moreover, in synergy with standard therapy, the adoption of an appropriate diet could be recommended to improve the clinical manifestations of Psoriasis and reduce the incidence of comorbidities [108]. There is a two-way relationship between MS and Psoriasis. One can predispose a patient to the onset of the other and worsen its symptoms, as psoriatic inflammation can, for example, contribute to MS. Evaluating the metabolic profile of each patient and formulating personalized treatments to resolve metabolic disorders and organism deficiencies has the potential to improve the course of both diseases and lead to the general improvement of an individual’s health.
